# BCAT1 controls embryonic neural stem cells proliferation and differentiation in the upper layer neurons

**DOI:** 10.1186/s13041-023-01044-8

**Published:** 2023-06-21

**Authors:** Shukui Zhang, Jinyue Zhao, Cheng Zhao, Libo Su, Jianwei Jiao

**Affiliations:** 1 College of Life Sciences, Yantai University, Yantai, 264005 Shandong China; 2 State Key Laboratory of Stem Cell and Reproductive Biology, Institute of Zoology, Chinese Academy of Sciences, Beijing, 100101 China; 3 University of Chinese Academy of Sciences, Beijing, 100049 China; 4 Beijing Institute for Stem Cell and Regenerative Medicine, Institute for Stem Cell and Regeneration, Chinese Academy of Sciences, Beijing, 100101 China; 5 Qingdao University, Qingdao, 266071 China

**Keywords:** BCAT1, Cerebral cortex, Neural progenitor cells, Upper layer neurons

## Abstract

The regulation of neural stem cell (NSC) proliferation and differentiation during brain development is a precisely controlled process, with the production of different neuronal subtypes governed by strict timelines. Glutamate is predominantly used as a neurotransmitter by the subtypes of neurons in the various layers of the cerebral cortex. The expression pattern of BCAT1, a gene involved in glutamate metabolism, in the different layers of neurons has yet to be fully understood. Using single-cell data, we have identified seven different states of NSCs and found that state 4 is closely associated with the development of projection neurons. By inferring the developmental trajectory of different neuronal subtypes from NSC subsets of this state, we discovered that BCAT1 is involved in the regulation of NSC proliferation and differentiation and is specifically highly expressed in layer II/III and IV neurons. Suppression of BCAT1 through shRNA resulted in a reduction in NSC proliferation and an abnormal development of layer II/III and IV neurons. These findings provide new insights into the role of BCAT1 in the regulation of NSC behavior and neuronal development.

**Supplementary Information:**

The online version contains supplementary material available at 10.1186/s13041-023-01044-8.

## Introduction

The cerebral cortex is a complex structure in the brain [1] that plays a critical role in advanced functions such as learning, cognition, and memory [2].The detailed structure of the cerebral cortex is composed of a six-layered architecture in an inside-out pattern [3],and it contains numerous neurons and glial cells that arise from various neural progenitor cells (NPCs) [4]. Radial glial cells are particularly notable for their ability to produce a remarkable diversity of neurons and glial cells, depending on the stage of development and location of the brain [5, 6].

Despite the well-known fact that the cortex’s excitatory neurons use glutamate as a neurotransmitter [7], the mechanisms of glutamate generation in different subtypes of neurons remain unclear. In this study, we aimed to verify the expression patterns of different genes involved in glutamate synthesis using single-cell data. Our results demonstrate that BCAT1 is intrinsically required for layer II/III and IV neurons generated in NSCs proliferation and differentiation.

Branched-chain amino transferase (BCAT) is an enzyme responsible for the reversible ammonia of leucine, isoleucine, and valine, which are collectively known as branched-chain amino acids (BCAAs). Transamination of BCAAs is the first step in their catabolism and leads to the formation of branched A-ketoacids, which are then decarboxylated to form coenzyme A (CoA) [8, 9] derivatives. In the case of leucine, transamination leads to ketone isohexanoic acid, which is further metabolized to form ketone bodies acetoacetate ketone bodies, acetyl coenzyme a, which is subsequently oxidized by the tricarboxylic acid (TCA) cycle, leading to the production of glutamate [10]. BCAT is present in two subtypes, mitochondrial BCAT2 and cytoplasmic BCAT1. Although Bcat2 is widely expressed in most tissues, especially in skeletal muscle, digestive system tissue, and kidney, it has been reported that BCAT1 expression is limited to embryonic tissue, adult brain, ovary, placenta, and neurons of the peripheral nervous system [11].

The branched-chain amino acid (BCAA) pathway and high levels of BCAA transaminase 1 have recently been associated with aggressiveness in several cancer entities [12–17]. However, the expression patterns of BCAT1 in different neuronal subtypes in the cerebral cortex remain largely uncertain. To verify our analysis results, we downregulated its expression in mouse embryonic NCSs via in utero electroporation (IUE) by BCAT1 shRNA, and the experimental data showed that BCAT1 knockdown decreased the proliferation of apical progenitors and the numbers of layer II/III and IV neurons. Our findings suggest that BCAT1 is a crucial regulator of cortical neuron generation and may provide insights into the pathogenesis of various brain diseases.

## Methods

### ScRNA-seq reads alignment and quantification

Raw sequencing data were using Cell Ranger pipeline aligned and qualification, and the GRCm38 mouse reference genome. Cells were filtered to retain higher-quality (mitochondrial genes < 10%, genes detected > 500 per cell).

### Cell cycle analysis

We use Seurat packages contained G1/S phase and G2/M phase cell-cycle-related gene set by ‘CellCycleScoring’ function analyzed each cell G2/M And S phase scores.

### Clustering and annotation

The quantification values were normalized of the feature expression for total expression, multiplying this by a scale factor (10,000), and log + 1 transformation of the result. Detected the 2000 most variable genes use Seurat ‘FindVariableFeatures’ function, using the ‘vst’ methods. PCA (Using variable genes by ‘RunPCA’ function) and Louvain graph-based clustering (by ‘FindCluster’ function, data dimensionality reduction using top 30 PCA numbers, and the clustering resolution was 0.6). For alleviate technical variation between different batches, the top 30 signification principle components (PCs) were selected by using R packages Harmony. The cluster cells identity was assigned by manual annotation using Know marker genes.

### Trajectory analysis

We used R packages Monocle 2 to analyze apical progenitors pseudotime trajectories. The dimension was reduced using ‘DDRTree’ methods. We used Python packages Streamto analyze neurons pseudotime trajectories analysis .

### Mice

The pregnant ICR mice were purchased from Beijing Vital River Laboratory Animal Technology Co, Ltd. All mice were kept in the institute of Zoology, Chinese Academy of Sciences (CAS) according to SPF standard.

### In utero electroporation

First use electroporator make glass micropipette and trim to the right length. According to a dose of 10 μl/g 0.7% pentobarbital sodium injected to the IRC pregnant mice at E13.5 make it anesthetized, and then in the super clean bench using sterilized surgical scissors cut open the inside and outside skin. Recombinant plasmid maxed with Venus-GFP at a 3:1 mol fetal and fast green was microinjected into the brain ventricles. In the different time point killed the pregnant mice, put the fetal brains fixed in 4% paraformaldehyde (PFA) 24 h and dehydration with 30% sucrose at 4 °C for 24 h. And last put the brains cut into sections for further analysis.

### Immunostaining

The brains slices were fixed in 4% PFA, washed with PBST (1% Triton X-100 in 1 M PBS). And incubated with primary antibodies 12 h at 4 °C, secondary antibodies 1 h, DAPI fluorescence-labeling 3 min.

### BrdU labeling

For NPC proliferation and cell cycle analysis, BrdU (50 mg/ml) was injected to pregnant female mice for 24 h before collecting embryonic brains.

### Western blotting

Tissue or cells were lysed with RIPA with protease inhibitor and ultrasonic cracking on ice then centrifuged at 4 °C for 15 min to eliminate cell debris. The BCA methods was used for measuring the concentration of protein. The protein samples was loaded onto SDS-PAGE gel for electrophoresis, and transferred the bands onto nitrocellulose membranes. Then incubated with primary antibody at 4 °C for 12 h and secondary antibodies 1 h.

### Cell culture

293FT were cultured in DMED medium that contained 10% FBS, 1% Non-Essential Amino Acid Solution and 1% Penicillin Streptomycin (PS).

Lentiviral packages DNA and the core DNA was transfected into HEK293FT by GenEscortTMI (Wisegen, Nanjing, China). At 24, 48, 72 h post-transfection collected the supernatant that containing the virus.

Ahead of 6 h, plates were coated with Poly-D-Lysine (10 μg/ml, Sigma) and Laminin (10 μg/ml, Invitrogen, Carlsbad, CA, USA). And NSCs were isolated from E12.5 cortex and seeded in the plates. For 6-well-plates, per well seeded 2 million cells. For 24-well-plates, per well seeded 0.3 million cells. Cells were cultured in proliferation medium for 12 h and then changed into differentiation medium.

### RT-qPCR

The extraction of total RNA and reverse-transcription of complementary DNA were performed as previously described. Quantitative real-time PCR (RT-qPCR) was performed in a 20 µl reaction mixture using Super Real SYBR Green PreMix Plus kit (TIANGEN) on ABI 7500 real-time PCR system (Applied Biosystems).

### Antibodies

Anti-Nestin (MAB353, Millipore), Anti-Satb2 (ad51502, Abcam), Anti-Ctip2 (ab18465, Abcam), Anti-Bcat1 (13640-1-AP, Proteintch), Anti-Pax6 (AB2237, Millipore, Darmstadt, Germany), Anti-Caspase3 (CST 9664S).

### Plasmid construction

BCAT1 cDNA was acquired by reverse transcription of mice brain total RNA and subcloned into the PCDH-CAG-GFP-FLAG vector to generate the FLAG-tagged pCDH-BCAT1 plasmid. ShRNAs for the target genes were cloned into the pSicoR-GFP vector.

BCAT1-sh1 GCATATTCCAACGATGGAGAA.

BCAT1-sh2 CCTTCCAAAGCCCTACTCTTT.

BCAT1-sh3 GATGGGAGAAACCTCACATTA.

### Statistical analysis

Immunostaining brain slices were imaged by Zeiss LSM 880 confocal microscope and images were analyzed by ZEN2010 (Lecia) and Photoshop (Adobe Systems). For all experiments, statistical analysis was performed using GraphPad Prism with unpaired t-test. All Results presented mean values ± SEM.

## Results

### Cortex single-cell transcriptome

**Fig. 1:**
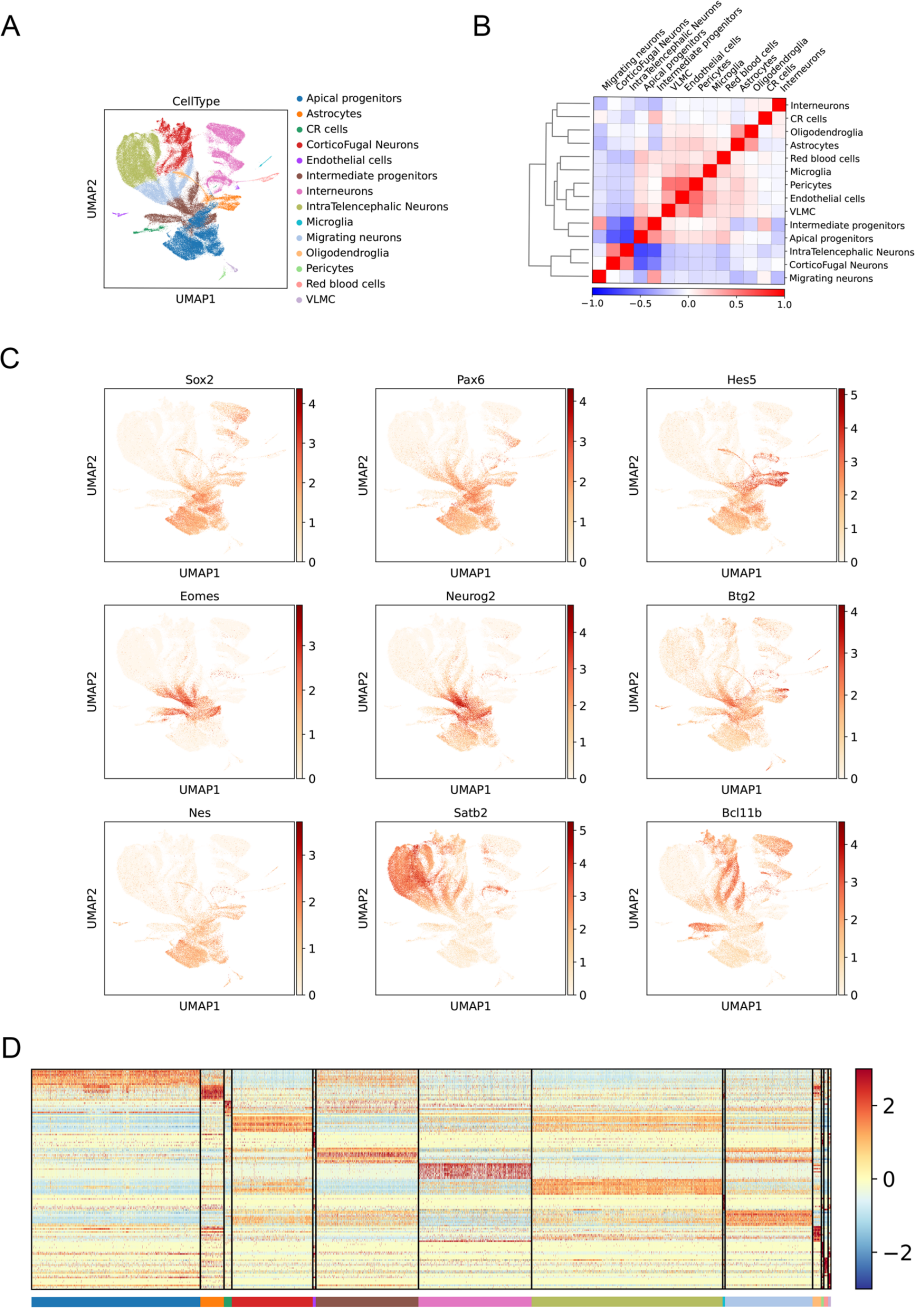
Cellular and molecular characteristics of the mouse cerebral cortex. **A** Visualization of mouse cortex cell clustering using UMAP. Cells are colored by cell-type assignment. **B** Different cell clusters correlation with the dendrogram. **C** UMAP visualizations colored by the expression of known marker genes for the selective cell population. Each dot represents one cell. **D** Heat map of top 10 DEGs between cell type clusters.

In our study, we aimed to comprehend the molecular features of mouse fetal brain development by analyzing single cell data from E10.5 to P4 mouse brain. The data was obtained from a publicly available database and underwent thorough quality control. Main cell types were annotated (Fig. 1A; Additional file 1: Table S1) and their correlations were analyzed (Fig. 1B), with neural precursor cells showing strong correlation and projective neurons displaying strong correlation as well. The composition of cells at different time points was found to be consistent with the stage of development (Additional file 2: Fig. S1A). Additionally, we confirmed the quality of the remaining cells by conducting quality control (Additional file 2: Fig. S1B). The annotation of cell types was validated by examining specific molecular signatures in each cluster from the differentially expressed genes (DEGs) with a log fold change greater than 0.5 (Fig. 1D).

By performing Uniform Manifold Approximation and Projection (UMAP) analysis, we identified the cell types as apical progenitors (AP; Sox2, Pax6, Hes5) and intermediate progenitors (IP; Eomes, Neurog2, Btg2). During the neuron genesis stage, we detected migrating neurons (Nrp1) (Fig. 1C) and different subtypes of excitatory neurons, including up-layer neurons (Satb2) and deep-layer neurons (Bcl11b) using known markers. These subtypes included layer 2&3 neurons, layer 4 neurons, layer 5&6 neurons, corticothalamic and subcerebral projecting neurons (CThPN and SCPN), layer 6b neurons, and near-projecting Tshz2-positive neurons (Fig. 1C). Ventrally generated inhibitory interneurons (Dlx2) were also identified (Additional file 2: Fig. S1C).

During the glial cell genesis stage, we detected oligodendrocyte precursor cells (OPC; Olig2) and astrocytes (Apoe). Additionally, we identified microglial cells (Aif1), red blood cells (Car2), endothelial cells (Cldn5), pericytes (Cspg4), and vascular and leptomeningeal cells (Col1a1) (Additional file 2: Fig. S1C).

### Developmental lineage of neural stem cells

**Fig. 2:**
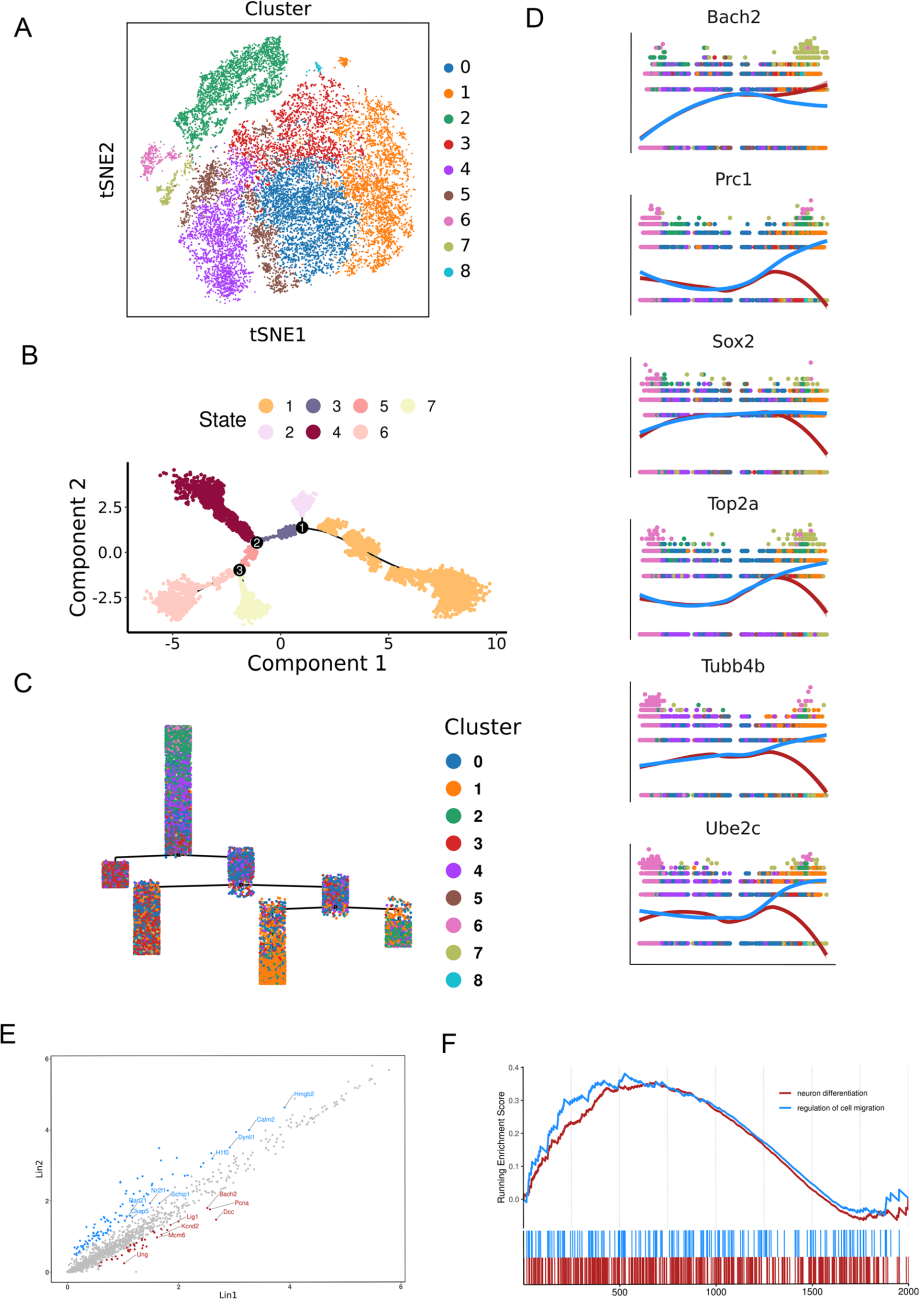
Lineage of neural precursor cells. **A** Clustering of apical progenitors from E10.5 to E17.5 visualized by t-SNE embedding. **B** Seven pseudotime state of apical progenitors by Monocle2. **C** The distribution of different cell clusters in seven pseudotime state. **D** Expression levels of apical progenitor genes along with pseudotime. The red and blue lines represent Lineage 2- State 4 and Lineage 2- State 6 and 7 cells. **E** Volcano plot of differential expressions of genes from Lineage 2- State 4 and Lineage 2- State 6 and 7 cells. . **F** The GSEA of GO biological processes ranked genes from high to low by values of log2 fold change. Adjusted P < 0.05

Studies have shown that neurons, oligodendrocytes, and astrocytes are derived from distinct subtypes of neural stem cells. In this study, we aimed to characterize single cells grouped in the apical progenitor clusters of the mouse fetal cortex. Through t-distributed stochastic neighbor embedding (t-SNE) analysis, we identified nine distinct subclusters (Fig. 2A; Additional file 3: Table S2) and identified the correlations between these subtypes (Additional file 2: Fig. S2B). We also found that Cluster 2 consisted almost entirely of cells at the E12.5 stage (Additional file 2: Fig. S2A). Cluster 7 expressed high levels of Scl1a3, suggesting a potential role in glial cell development, while Cluster 8 expressed high levels of neurogenic-related genes Bcl11b and Neurog2, suggesting a correlation with neural development (Additional file 2: Fig. S2C and D).

To understand the developmental trajectory of these nine subclusters, we divided them into seven states (Fig. 2B; Additional file 4: Table S3) and reconstructed a lineage tree using Monocle 2 (Fig. 2C). We found that subclusters 2 and 6 were at the top of the tree, and Cluster 4 was mainly spread out in the inflection point in a genealogical shift. These results suggest that Clusters 2 and 4 represent an earlier developmental status and the pre-transition state of different subtypes, which correlates with the main component of E12.5 cells in Cluster 2 and E12.5, E13.5, E15.5 cells' uniform distribution in Cluster 4, 5 and 6 (Additional file 2: Fig. S2A). Furthermore, we found that Clusters 7 and 8 were almost exclusively in State 4, so we analyzed the different gene expression patterns between Lineage 2- State 4 and Lineage 2- State 6 and 7. We observed increased expression of Bach2 in Lineage 2- State 4, and Prc1, Sox2, Top2a, Tubb4b, and Ube2c showed increased expression in Lineage 2- State 6 and 7 (Fig. 2D).

To further understand the discrepancy between Lineage 2-State 4 and Lineage2-State 6 and 7, we performed differential analysis and identified specific expression genes between the two cell lineages (adjusted P < 0.05 and log fold change > 0.25) (Fig. 2E). We then conducted gene set enrichment analysis (GSEA) of these genes and ranked them from high to low using values of log2 fold change. We found that Lineage 2-State 4 specifically expressed genes that were functionally related to neuron differentiation, while Lineage 2-State 6 and 7 specifically expressed genes that were functionally related to the regulation of cell migration (Fig. 2F). Our results suggest that in the anaphase of neural stem cell proliferation (Additional file 2: Fig. S2E), a precursor cell produces two seed generations, one that tends to produce neurons and the other that tends to migrate.

In conclusion, our study provides a comprehensive characterization of single cells grouped in the apical progenitor clusters of the mouse fetal cortex. We identified distinct subclusters, developmental trajectories, and specific expression genes that suggest a potential role in neuron differentiation and cell migration. Our results contribute to the understanding of neural stem cell proliferation and differentiation and may have implications for the development of new therapeutic approaches for neurological disorders.

### BCAT1 is mainly expressed in layer II/III and IV neurons

**Fig. 3:**
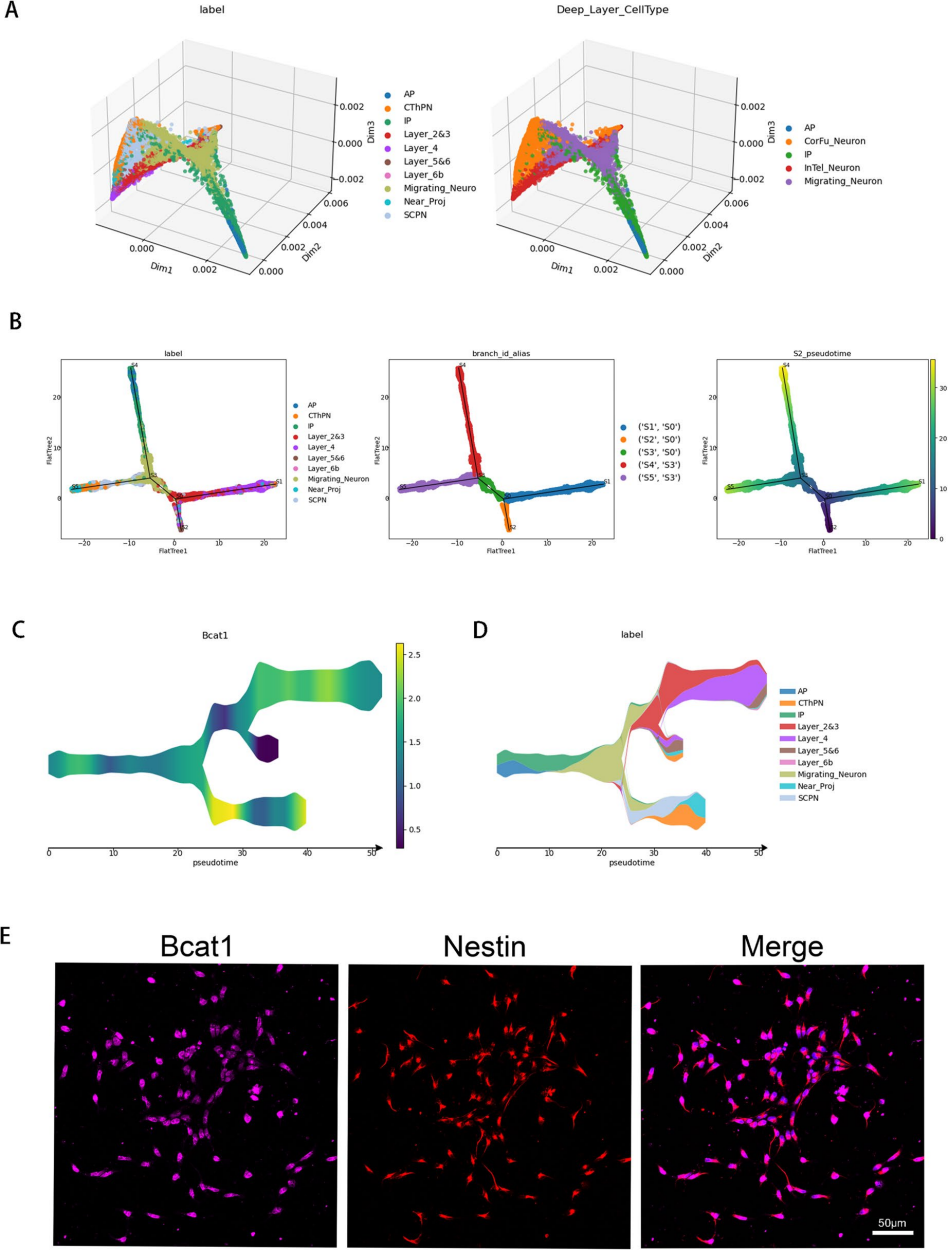
Expression of Bcat1 in the developmental trajectory of neurons. **A** Visualization of different cell types using Stream in 3D. **B** Flat tree plot constructed from apical progenitors, intermediate progenitors and excitatory neurons. Labeled by cell type, branch id and pseudotime. **C** Stream plot of Bcat1 gene expression. **D** Stream plot, colored by cluster labels inferred. **E** Bcat1 co-stained with the NSCs markers of NESTIN. NSCs were isolated from the E12.5 mice brains and cultured in the proliferative medium for 24 h. Scale bar represents 50 μm

In the field of neuron development, the proximity between different neuron subtypes is often visualized in a three-dimensional scatter plot (Fig. 3A), which displays the cells' positions in the MLLE space, fitted with a principal graph using STREAM [18]. In order to more intuitively and conveniently represent the trajectories of these cells in a two-dimensional plane, a Flat tree plot was constructed, preserving the lengths of tree branches in the MLLE space (Fig. 3B).

Our analysis revealed that apical progenitor cells and intermediate progenitor cells predominantly appear on the S4-S3 branch, while corticothalamic, subcerebral PN (CThPN and SCPN) and near-projecting neurons are primarily located on the S3-S5 branch. Layer II/III and IV neurons are primarily found on the S0-S1 branch, and layer 5 and 6 neurons are primarily located on the S0-S2 branch. Further, we identified differential genes in these different branches (Additional file 2: Fig. S3A-F).

One gene of interest that has been extensively studied in the context of cancer, BCAT1, is also involved in glutamate metabolism in excitatory neurons in the cerebral cortex. Based on our analysis, we found that BCAT1 is highly expressed in layer II/III and IV neurons, as well as in partially migrating neurons. However, it is less expressed in deep neurons (Fig. 3C and D). These results are consistent with our findings, as we observed that BCAT1 is co-expressed with the neural stem cell marker Nestin (Fig. 3E).

### BCAT1 regulates neural progenitor cell proliferation

**Fig. 4:**
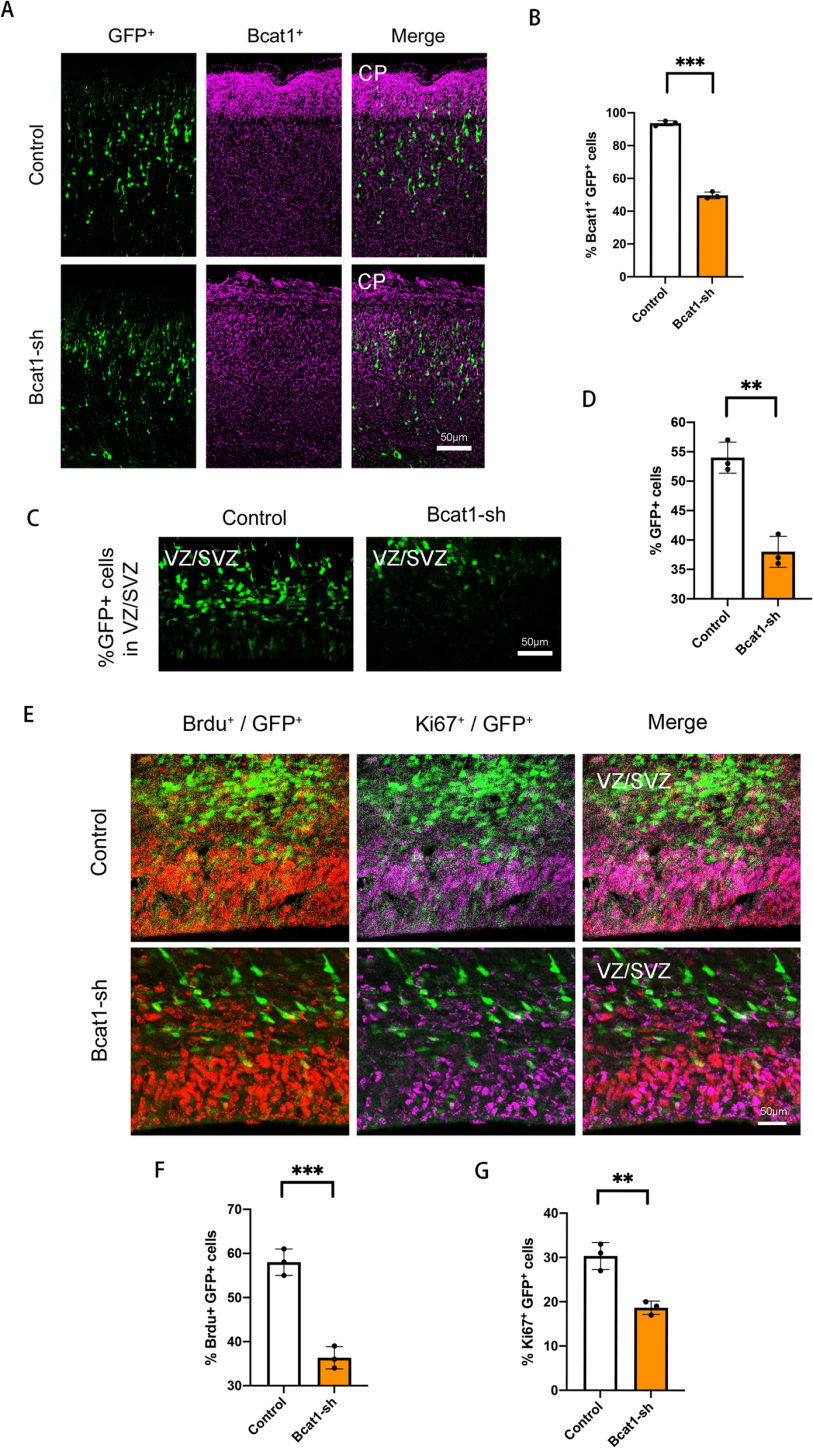
Bcat1 regulates the proliferation of the NSCs. **A, B** The percentage of Bcat1^+^GFP^+^ cells are decreased in Bcat1 knockdown brains. The mouse was electroporated at E13.5 and killed at E18.5. The bar graph shows the percentage of Bcat1^+^GFP^+^ cells relative to the control Bcat1^+^GFP^+^ cells (n = 3 independent experiments; ***P < 0.001; bars represent mean ± S.E.M). The scale bar represents 50 μm. **C, D** The GFP^+^ cells are decreased in Bcat1 knockdown brains. The mouse brain was electroporated at E13.5 and BrdU (50 mg/kg) was injected 24 h prior to killing at E15.5. (n = 3 independent experiments; **P < 0.01; bars represent mean ± S.E.M). The scale bar represents 50 μm. **E–G** The percentage of GFP^+^ BrdU^+^ cells and GFP^+^ Ki67^+^ cells are decreased in Bcat1 knockdown brains. The mouse brain was electroporated at E13.5 and BrdU (50 mg/kg) was injected 24 h prior to killing at E15.5. The bar graph shows the percentage of GFP^+^ BrdU^+^ cells and GFP^+^ Ki67^+^ cells relative to the total GFP-positive cells (n = 3 independent experiments; **P < 0.01; bars represent mean ± S.E.M). The scale bar represents 20 μm

To investigate the function of the *Bcat1* gene in neural stem cells (NSCs), we utilized short hairpin RNA (shRNA) to knockdown BCAT1 expression in neural progenitor cells of the developing cortex in E13.5 mouse embryos via uterine electroporation (IUE). We collected the brains at E18.5 for subsequent phenotypic analysis. The efficiency of BCAT1 knockdown was confirmed by assessing BCAT1 expression, which showed a significant decrease with the introduction of BCAT1 shRNA (Fig. 4A and B). Furthermore, we observed a decrease in the expression of the neural stem cell marker Pax6 and the proliferation marker Ki67 in vitro after BCAT1 knockdown, indicating a potential role of BCAT1 in NSCs proliferation and differentiation (Additional file 2: Fig. S4A and B).

In vivo, we found that knockdown of BCAT1 resulted in significant changes in cell distribution compared to the control group. The number of GFP positive cells in the proliferating ventricular/subventricular zone (VZ/SVZ) was significantly decreased (Fig. 4C and D). To determine whether this decrease in GFP-positive cells in VZ/SVZ was due to a decrease in neural precursor cell proliferation, we injected BrdU 24 h before brain collection at E15.5. We observed a 20% reduction in BrdU and GFP double positive cells in the BCAT1 knockdown group (Fig. 4E and F), suggesting a decrease in neural precursor cell proliferation. We also used immunostaining with the proliferative marker Ki67 to confirm this result. The proportion of double positive cells showed a significant decrease in the percentage of cells that were positive for Ki67 and labeled with GFP in the BCAT1 knockdown group (Fig. 4E and G). Finally, we measured the number of apoptotic cells and found that BCAT1 knockdown did not cause a significant increase in apoptosis (Additional file 2: Fig. S4C and D). Taken together, our findings suggest that BCAT1 is important for NSCs proliferation and may play a role in regulating the development of layer II/III and IV neurons in the cerebral cortex.

### BCAT1 deletion leads to loss of neurons

**Fig. 5:**
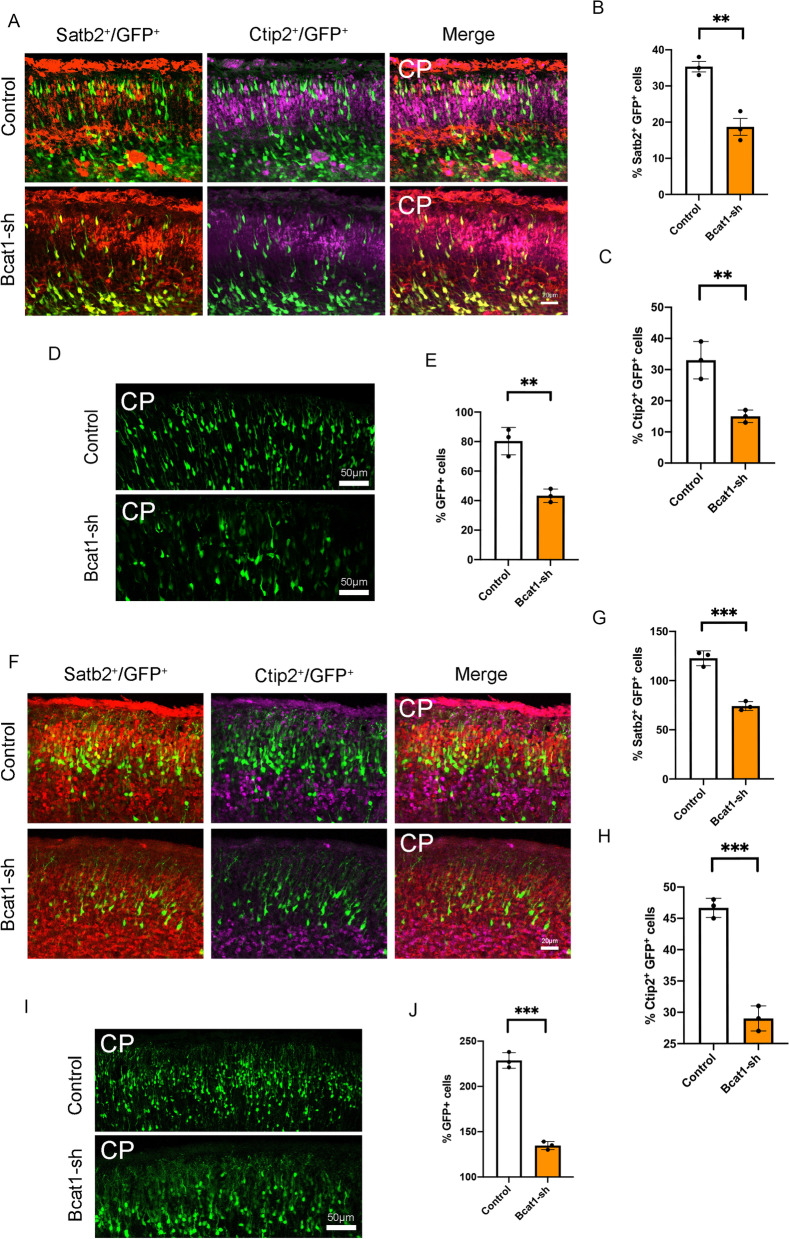
Bcat1 Knockdown causes the abnormal neuronal development. **A–C** The percentage of GFP^+^ Satb2^+^ cells and GFP^+^Ctip2^+^ cells are decreased in Bcat1 knockdown brains. The mouse was electroporated at E13.5 and killed at E16.5. The bar graph shows the percentage of GFP^+^ Satb2^+^ cells and GFP^+^ Ctip2^+^ cells relative to the control GFP^+^ Satb2^+^ cells and GFP^+^ Ctip2^+^ cells (n = 3 independent experiments; **P < 0.01; bars represent mean ± S.E.M). The scale bar represents 20 μm. **D, E** The GFP + cells are decreased in Bcat1 knockdown brains. The mouse brain was electroporated at E13.5 and killing at E16.5. (n = 3 independent experiments; **P < 0.01; bars represent mean ± S.E.M). The scale bar represents 50 μm. **F–H** The percentage of GFP^+^ Satb2^+^ cells and GFP^+^ Ctip2^+^ cells are decreased in Bcat1 knockdown brains. The mouse brain was electroporated at E13.5 and killing at E18.5. The bar graph shows the percentage of GFP^+^ Satb2^+^ cells and GFP^+^ Ctip2^+^ cells relative to the control GFP^+^ Satb2^+^ cells and GFP^+^ Ctip2^+^ cells (n = 3 independent experiments; **P < 0.01; bars represent mean ± S.E.M). The scale bar represents 20 μm. **I, J** The GFP^+^ cells are decreased in Bcat1 knockdown brains. The mouse brain was electroporated at E13.5 and killing at E18.5. (n = 3 independent experiments; **P < 0.01; bars represent mean ± S.E.M). The scale bar represents 50 μm

To directly investigate whether BCAT1 knockdown affects the differentiation of II/III and IV layers of neurons, we collected brains at E16.5 after E13.5 electroporation of the knockdown plasmid performed immunofluorescent staining for electroperforated brain sections with the neuronal markers Satb2 and Ctip2. Our results showed that the proportion of GFP-Satb2 double-positive cells was significantly reduced in the BCAT1 knockout group (Fig. 5A and B). Similarly, we counted the number of Ctip2-labeled neurons and found that the proportion of Ctip2-GFP double-positive cells was also significantly reduced (Fig. 5A and C). These findings were consistent with the significantly reduced phenotype of GFP-positive cells (Fig. 5D and E).

To further verify the impact of BCAT1 on the production of layers II/III and IV neurons, we collected brains at E18.5 after E13.5 electroporation of the knockdown plasmid. As expected, the reduction in layer II/III and IV neurons was more pronounced in the BCAT1 knockout mice than in the control group (Fig. 5F–H). Similarly, the number of GFP-positive cells was significantly reduced (Fig. 5I and J). We also tested whether BCAT1 knockdown impaired the migration of neurons by collecting brains at P0 after E15 electroporation of the knockdown plasmid. The results showed that the proportion of migration was not affected (Additional file 2: Fig. S4E and F). Additionally, we performed RT-qPCR to detect the expression of Cux1 and Dcx in cultured NPCs. Compared with the control group, BCAT1 knockdown significantly reduced the expression of Cux1 and Dcx (Additional file 2: Fig. S5A and B). These findings indicate that BCAT1 knockdown can significantly affect the development of layers II/III and IV neurons.

## Discussion

Neuronal signaling and regulation of excitatory and inhibitory signaling are mediated and regulated through neurotransmitters and their receptors in the brain [19–21]. Despite being a crucial excitatory neurotransmitter in the central nervous system, the recognition of glutamate as a neurotransmitter has been a slow process, partially due to its abundance in brain tissue and its involvement in multiple metabolic pathways [22–24]. Approximately 5–15 mmol of glutamate can be found per kg of brain tissue [23]. Glutamate has been shown to play a critical role in behavioral functions such as memory and learning and is considered the main excitatory neurotransmitter in the central nervous system [26–28]. In addition to its functions in synaptic transmission and neuronal plasticity, glutamate also holds a key position in forebrain neurogenesis, regulating cell survival, proliferation, migration, and differentiation [29].

Multiple lines of evidence suggest that glutamate promotes neural growth and survival during development [29]. Our study provides novel insight into the role of BCAT1 in NSC proliferation in vivo. Utilizing single-cell data analysis during mouse cortical development, we were able to identify neural progenitor cells in apical progenitor cells, and further trace the developmental trajectory of different subtypes of neurons in the cerebral cortex. Our analysis of BCAT1 expression levels in this developmental locus revealed dynamic expression patterns, which varied across different cell types. Immunostaining on electroperforated brain sections at the stage of NSC proliferation showed co-labeling of BCAT1 with the NSC marker Nestin, suggesting that the proliferation and differentiation of NSCs require the expression of BCAT1. Our IUE results showed that knockdown of BCAT1 at E15.5 resulted in decreased proportions of GFP-positive NSCs and BrdU/Ki67 positive proliferative NSCs, indicating weakened proliferation and differentiation abilities of NSCs upon BCAT1 knockdown. At E18.5, we observed a decrease in the proportion of Satb2 and Ctip2 labeled neurons in the upper layer, demonstrating that the early effect of BCAT1 knockdown on NSCs can persist until later stages of development. However, the number of lower layer neurons remained unchanged, highlighting the subtype-specific effect of BCAT1 on neuronal development.

In conclusion, our findings provide new insights into the role of BCAT1 in the development of specific neuronal subtypes and contribute to our understanding of the regulation of glutamate metabolism in the development of neuronal subtypes.

## Supplementary Information

**Additional file 1. Table S1.** Each cell corresponds to the period and cell type in Figure 1.

**Additional file 2.** Supplement Figure S1-S5.

**Additional file 3. Table S2.** Each cell corresponds to the Cluster ID in Figure 2.

**Additional file 4. Table S3.** Each cell corresponds to the State ID in Figure 2.

## Data Availability

The datasets used and/or analyzed during the current study are available from the corresponding author on reasonable request. Data and materials availability: All sequencing-derived raw RNA data reported in this paper are available from GEO (http://www.ncbi.nlm.nih.gov/geo). The accession number for the RNA-seq data is GEO: GSE153162. A detailed description of the computational processing and parameters is provided in method details.

## Abbreviations

NSCs: Neural stem cells
NPCs: Neural progenitor cells
BCAT: Branched-chain amino transferase
BCAAs: Branched-chain amino acids
CoA: Coenzyme A
TCA: Tricarboxylic acid
E13.5: Embryonic day 13.5
P0: Postnatal day 0
IUE: In utero electroporation
RT-qPCR: Quantitative real-time PCR
t-SNE: T-distributed stochastic neighbor embedding
UMAP: Uniform Manifold Approximation and Projection
shRNA: Short hairpin RNA
VZ/SVZ: Ventricular/subventricular zone
