# Supplementary material for: BCAT1 controls embryonic neural stem cells proliferation and differentiation in the upper layer neurons
Source: Mol Brain. 2023 Jun 21;16:53. doi: 10.1186/s13041-023-01044-8 (PMC10283284; doi:10.1186/s13041-023-01044-8)
Supplement: Supplementary file 2 — Additional file 2. Supplement Figure S1-S5. [file 13041_2023_1044_MOESM2_ESM.pdf]

FigS1

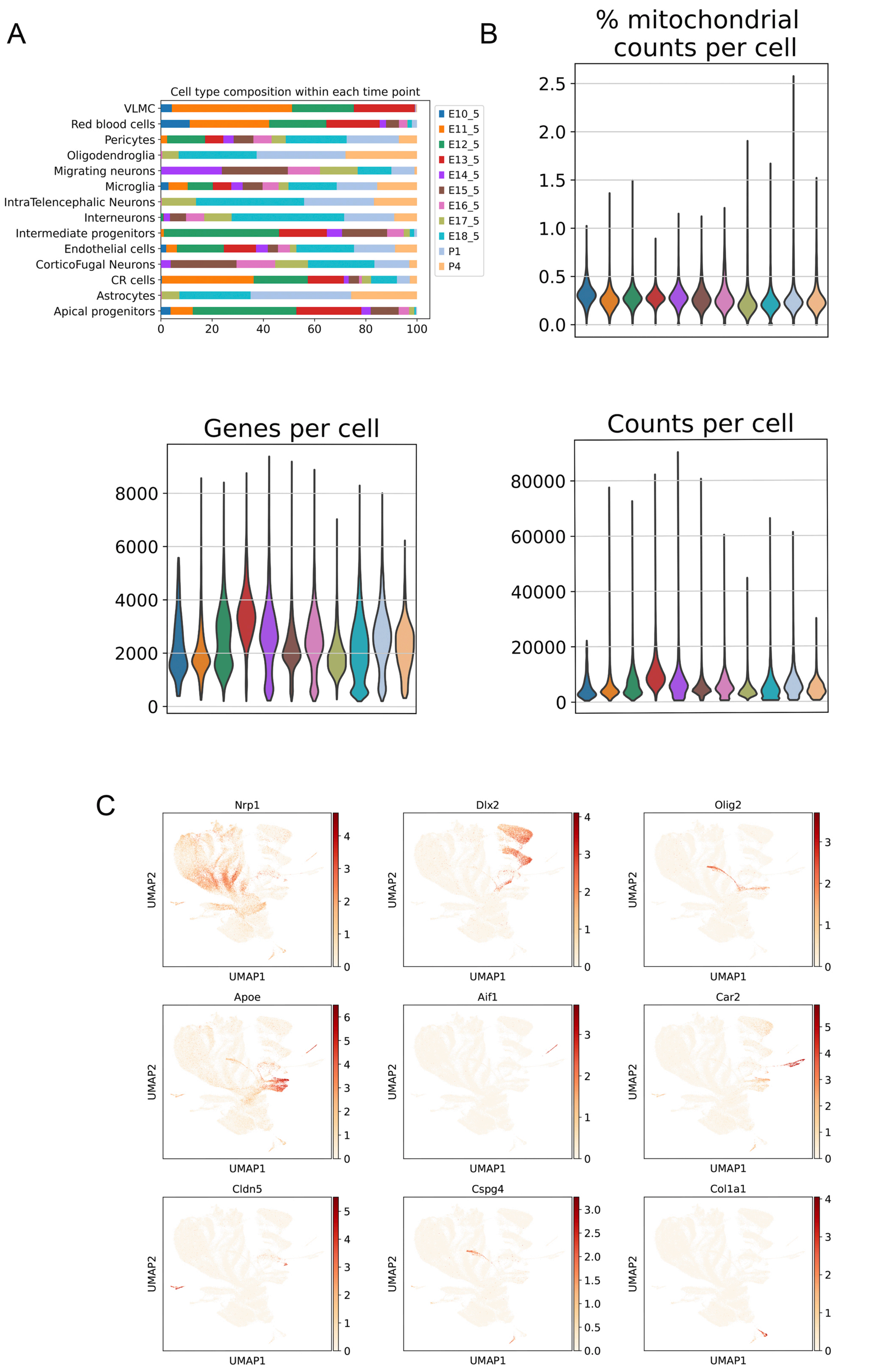
A
B
Cell type composition within each time point
VLMC
Red blood cells
Pericytes
Oligodendroglia
Migrating neurons
Microglia
IntraTelencephalic Neurons
Interneurons
Intermediate progenitors
Endothelial cells
CorticoFugal Neurons
CR cells
Astrocytes
Apical progenitors
0
20
40
60
80
100
E10_5
E11_5
E12_5
E13_5
E14_5
E15_5
E16_5
E17_5
E18_5
P1
P4
% mitochondrial counts per cell
0.0
0.5
1.0
1.5
2.0
2.5
Genes per cell
0
2000
4000
6000
8000
Counts per cell
0
20000
40000
60000
80000
C
Nrp1
Dlx2
Olig2
Apoe
Aif1
Car2
Cldn5
Cspg4
Col1a1
UMAP1
UMAP2

Additional file 2: Figure S1.

Classification of cell types in scRNA-seq data.

(A).Proportion of cells corresponding to the different cell types present in each time point.

(B).Number of genes, number of mRNA molecules (counts) and percentage of mitochondrial counts per cell in each time point.

(C).UMAP visualization of cells type marker genes expression levels.

FigS2

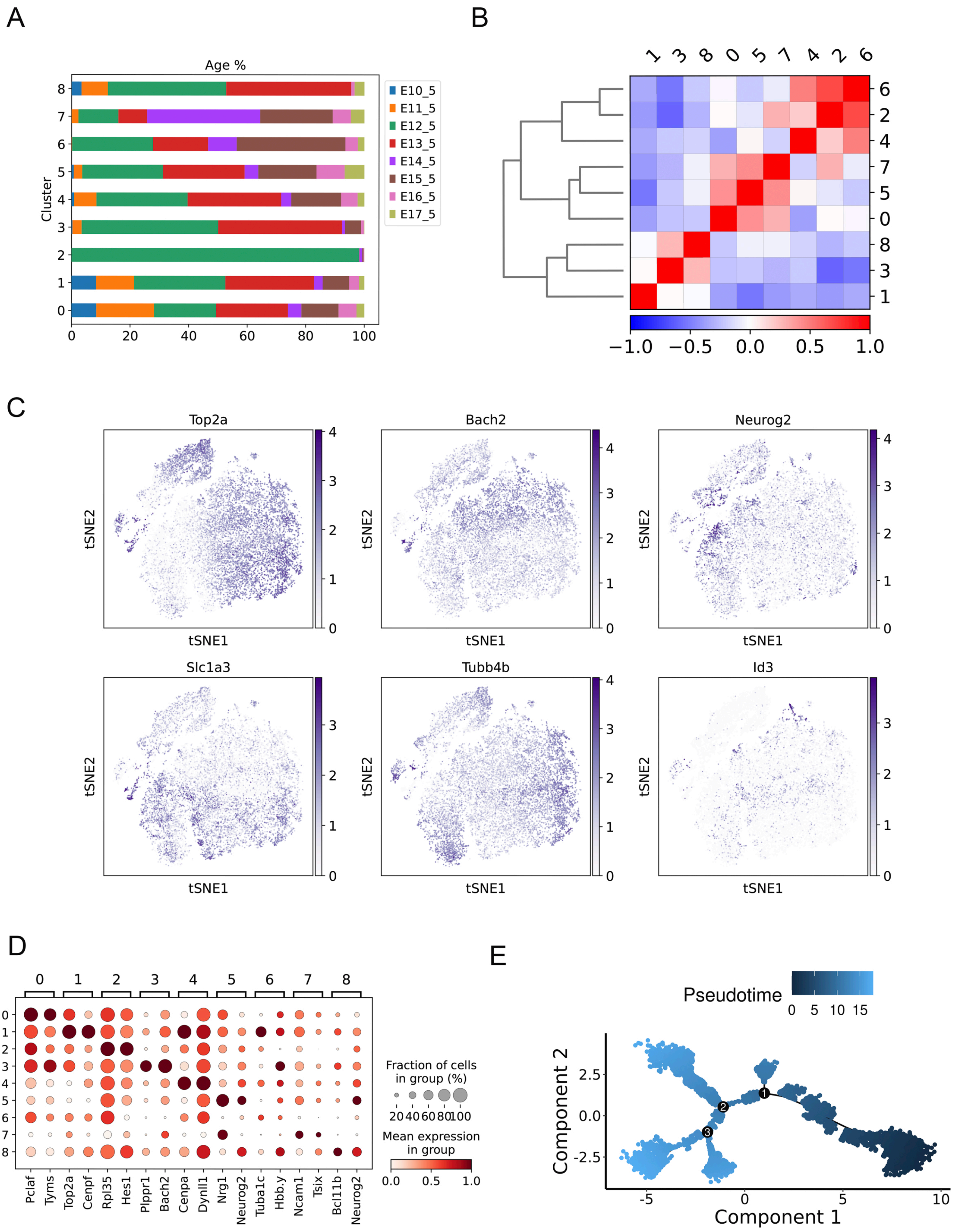
A
Age %
Cluster
E10_5
E11_5
E12_5
E13_5
E14_5
E15_5
E16_5
E17_5
0
20
40
60
80
100
B
1 3 8 0 5 7 4 2 6
6
2
4
7
5
0
8
3
1
−1.0 −0.5 0.0 0.5 1.0
C
Top2a
Bach2
Neurog2
Slc1a3
Tubb4b
Id3
tSNE1
tSNE2
D
Fraction of cells in group (%)
20 40 60 80 100
Mean expression in group
0.0 0.5 1.0
Pclaf
Tyms
Top2a
Cenpf
Rpl35
Hes1
Plppr1
Bach2
Cenpa
Dynll1
Nrg1
Neurog2
Tuba1c
Hbb.y
Ncam1
Tsix
Bcl11b
Neurog2
E
Pseudotime
0 5 10 15
Component 1
Component 2
2.5
0.0
-2.5
-5
0
5
10

Additional file 2: Figure S2.

Characteristics of neural progenitor cell subtypes

(A).Number of neural progenitor cell subtypes per time point.

(B).Neural progenitor cell subtype correlation heatmap

(C).UMAP visualization of neural progenitor cell subtype marker genes expression levels.

(D).Neural progenitor cell subtype specific gene dot plot

(E).Pseudo-temporal distribution of neural progenitor cells

FigS3

A

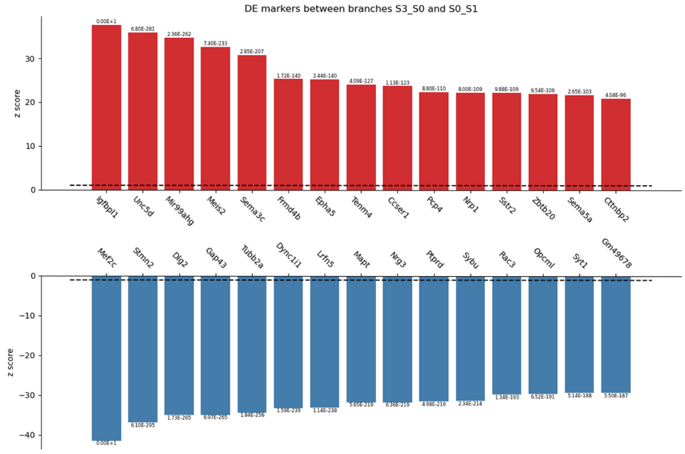

B

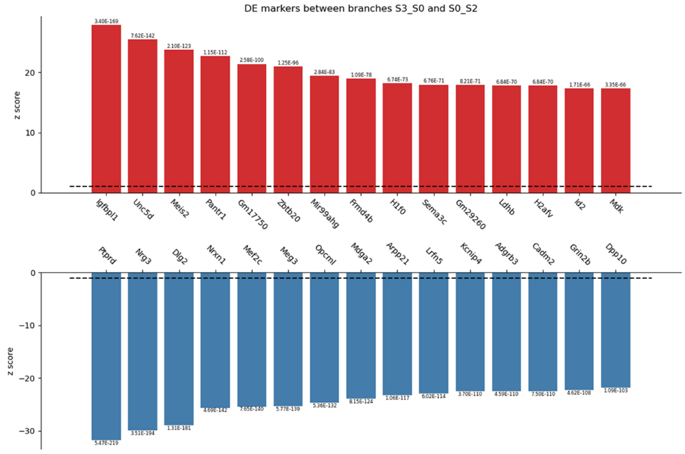

C

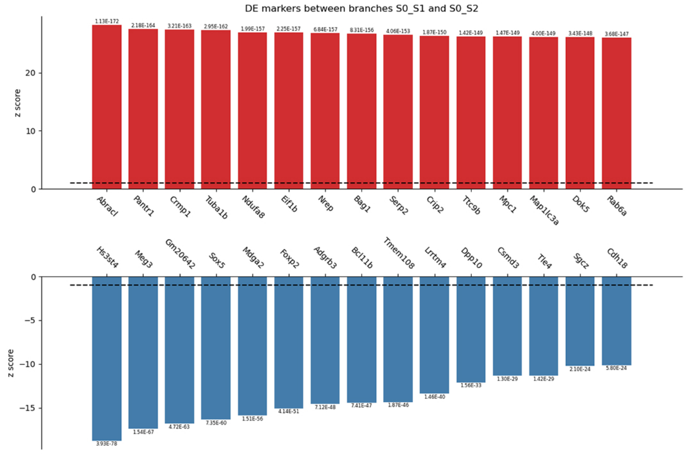

D

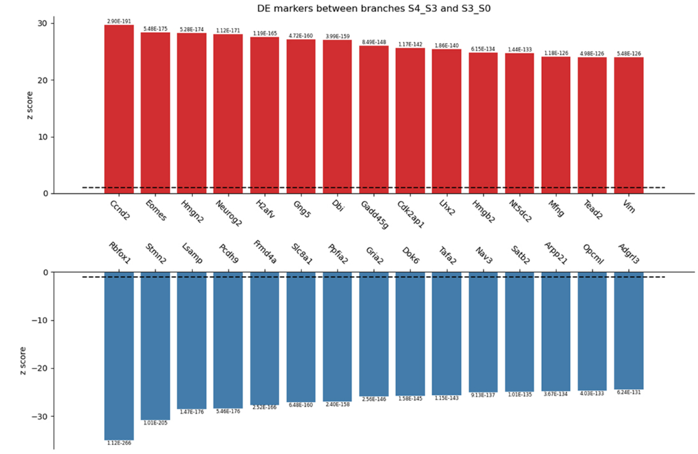

E

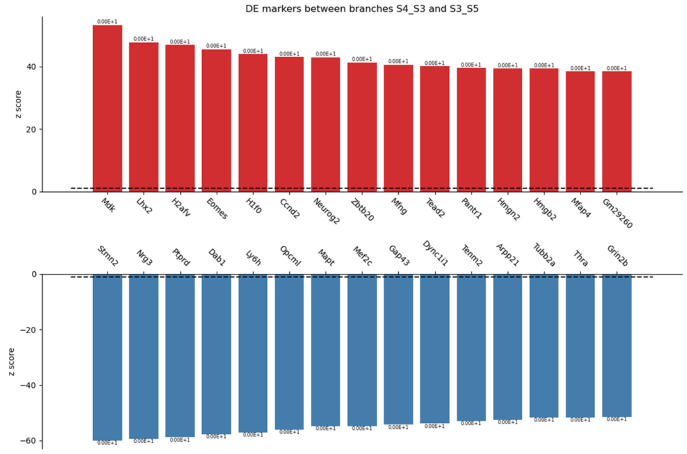

F

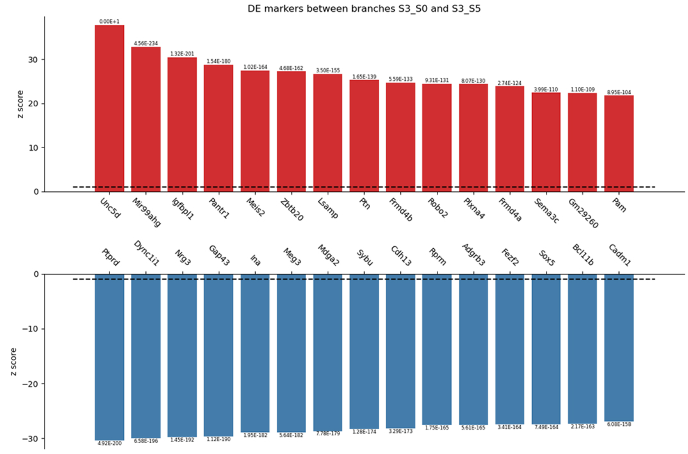

Additional file 2: Figure S3.

Characteristics of neural progenitor cell subtypes

(A-F). Differentially expressed genes between different branches of cell differentiation locus

FigS4

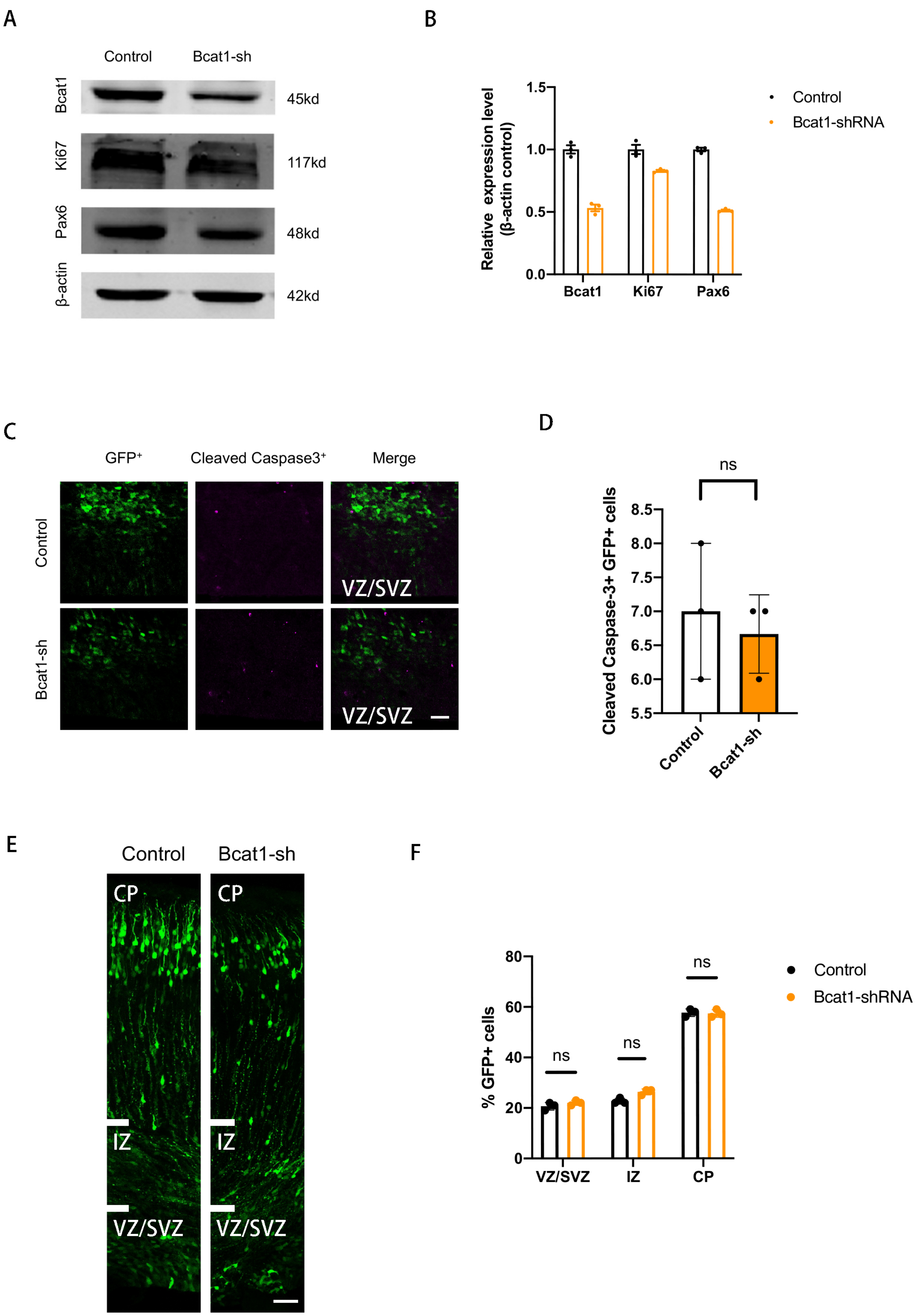
A
Control
Bcat1-sh
Bcat1
Ki67
Pax6
β-actin
45kd
117kd
48kd
42kd
B
Relative expression level
(β-actin control)
1.5
1.0
0.5
0.0
Bcat1
Ki67
Pax6
Control
Bcat1-shRNA
C
GFP+
Cleaved Caspase3+
Merge
Control
Bcat1-sh
VZ/SVZ
VZ/SVZ
D
ns
Cleaved Caspase-3+ GFP+ cells
8.5
8.0
7.5
7.0
6.5
6.0
5.5
Control
Bcat1-sh
E
Control
Bcat1-sh
CP
CP
IZ
IZ
VZ/SVZ
VZ/SVZ
F
% GFP+ cells
80
60
40
20
0
ns
ns
ns
VZ/SVZ
IZ
CP
Control
Bcat1-shRNA

Additional file 2: Figure S4.

Bcat1 knockdown did not induce apoptosis or decrease the ability of neuron migration

(A,B). Protein level of neural stem cell marker and proliferation mater, including Pax6 and Ki67 are reduced in the Bcat1 knockdown NSCs versus the control (n=3 independent experiments; bar represents mean ± S.E.M; **P<0.01; β-ACTIN served as loading control).

(C,D). Bcat1 knockdown did not cause an increase in the proportion of GFP+ Cleaved Caspase-3+ cells compared with the control group. The mouse was electroporated at E13.5 and killed at E15.5. The bar graph shows the percentage of GFP+ Cleaved Caspase-3+ cells relative to the control GFP+ Cleaved Caspase-3+ cells (n=3 independent experiments; *P<0.05; bars represent mean ± S.E.M). The scale bar represents 50μm.

(E,F). Compared with the control group, knockdown of Bcat1 did not cause a reduction in cell migration ability. The mouse was electroporated at E15 and killed at P0. The bar graph shows the percentage of GFP+ cells in VZ/SVZ，IZ,CP relative to the total GFP+ cells (n=3 independent experiments; *P<0.05; bars represent mean ± S.E.M). The scale bar represents 50μm.

FigS5

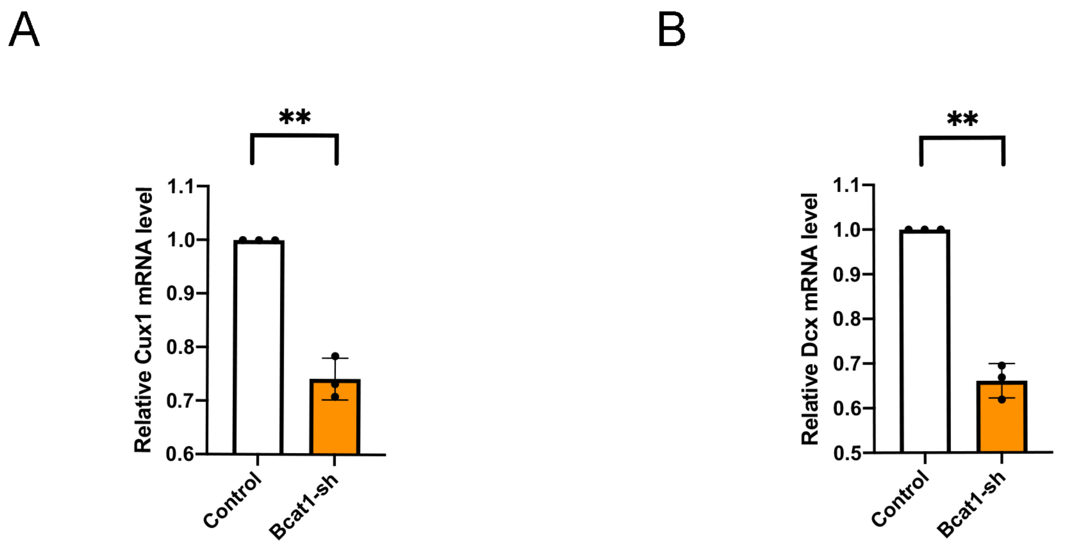
A
B
**
Relative Cux1 mRNA level
1.1
1.0
0.9
0.8
0.7
0.6
Control
Bcat1-sh
**
Relative Dcx mRNA level
1.1
1.0
0.9
0.8
0.7
0.6
0.5
Control
Bcat1-sh

Additional file 2: Figure S5.

Bcat1 knock-down results in lower levels of genes associated with upper layer neurons (A,B).Realtime-PCR analysis of Cux1 and Dcx gene expression in Bcat1 knockdown NSCs versus the control (n=3 independent experiments; bar represents mean ± S.E.M; **P<0.01).
